# Case Report: Acute Myocarditis Due to PD-L1 Inhibitor Durvalumab Monotherapy in a Patient With Lung Squamous Cell Carcinoma

**DOI:** 10.3389/fmed.2022.866068

**Published:** 2022-06-21

**Authors:** Bo Zhou, Manxiang Li, Tianjun Chen, Jianqing She

**Affiliations:** ^1^Respiratory and Critical Care Medicine, The First Affiliated Hospital of Xi'an Jiaotong University, Xi'an, China; ^2^Cardiology Department, The First Affiliated Hospital of Xi'an Jiaotong University, Xi'an, China

**Keywords:** durvalumab, PD-L1 inhibitor, myocarditis, empyema, lung squamous cell carcinoma

## Abstract

**Background:**

Durvalumab, as a PD-L1 inhibitor, is commonly used for the treatment of various cancers. Adverse events associated with the therapy include hepatitis, nephritis, dermatitis, and myocarditis. Especially, myocarditis as an adverse event after PD-L1 inhibitor therapy is characterized for its low incidence and high mortality.

**Case Summary:**

Here we present a rare case of a 67-year-old male with lung squamous cell carcinoma complicated with empyema who experienced myocarditis after only PD-L1 inhibitor durvalumab monotherapy. He presented with markedly decrease left ventricular ejection fraction, elevated Natriuretic peptide BNP, Troponin T, Troponin I, ESR, CRP and interleukin-6. The electrocardiogram showed sinus tachycardia, low voltage of limb leads, T wave inversion in anterior waves and V1-V3 QS type. Myocardial injury occurred in a short period and quickly returned to normal after glucocorticoids therapy.

**Conclusion:**

This case report is of clinical value for the treatment of PD-L1 related myocarditis.

## Introduction

Immune checkpoint inhibitors (ICIs) has brought revolutionary breakthroughs to tumor therapy (1–3). However, with the increased utilization of ICIs, the associated adverse events are becoming more and more recognized. ICIs treatment was originally intended to enhance the body's immunity. But in some cases, the immune system was overcorrected, causing its own immune cells to attack the host tissues and organs, which led to the corresponding ICIs related adverse reactions (irAEs). Specifically, myocarditis as one of the irAEs is characterized by its low incidence and high mortality, which deserves immediate recognition (4, 5). Previously, cases of myocarditis and fatal heart failure have been reported among patients with lung cancer treated with ICIs, especially among those receiving programmed cell death 1 (PD-1) inhibitor with or with chemical therapy as combination treatment (6, 7). Nevertheless, clinical evidence regarding myocarditis after programmed cell death ligand-1 (PD-L1) inhibitor treatment are still lacking due to its low incidence. Here, we presented the case of acute immune-mediated myocarditis associated with a PD-L1 inhibitor durvalumab monotherapy in a patient with lung squamous cell carcinoma complicated with empyema.

## Case Report

A 67-year-old male patient was admitted to the hospital with new onset fever, chest pain and dyspnea for 7 days and previous diagnose of right lung squamous cell carcinoma. His previous medical history was notable for right lung squamous cell carcinoma stage IV (T_3_N_3_M_1_) complicated with mediastinal lymph nodes and liver metastasis 1 year before. Cardiac and pulmonary function was normal at that time. Four cycles of chemotherapy with paclitaxel-cisplatin regimen was initiated but afterwards stopped due to 2019 coronavirus outbreak. Ten months thereafter, he was admitted to the hospital because of right massive pleural effusion. Twice bacterial culture of pleural effusion displayed *Prevotella nigrescens*, indicating right lung squamous cell carcinoma complicated with empyema. With subsequent treatment of meropenem as the anti-bacterial agent, his symptoms were relieved and his temperature was normal. As a result, chemotherapy was discontinued and replaced with PD-L1 immune checkpoint inhibitor, durvalumab monotherapy for four cycles (500 mg intravenous drip). And he presented with the symptom of fever, chest pain and dyspnea 7 days after last cycle of durvalumab. Moreover, the previous medical, family, and psychosocial history as well as genetic information showed nothing special.

**Figure 1:**
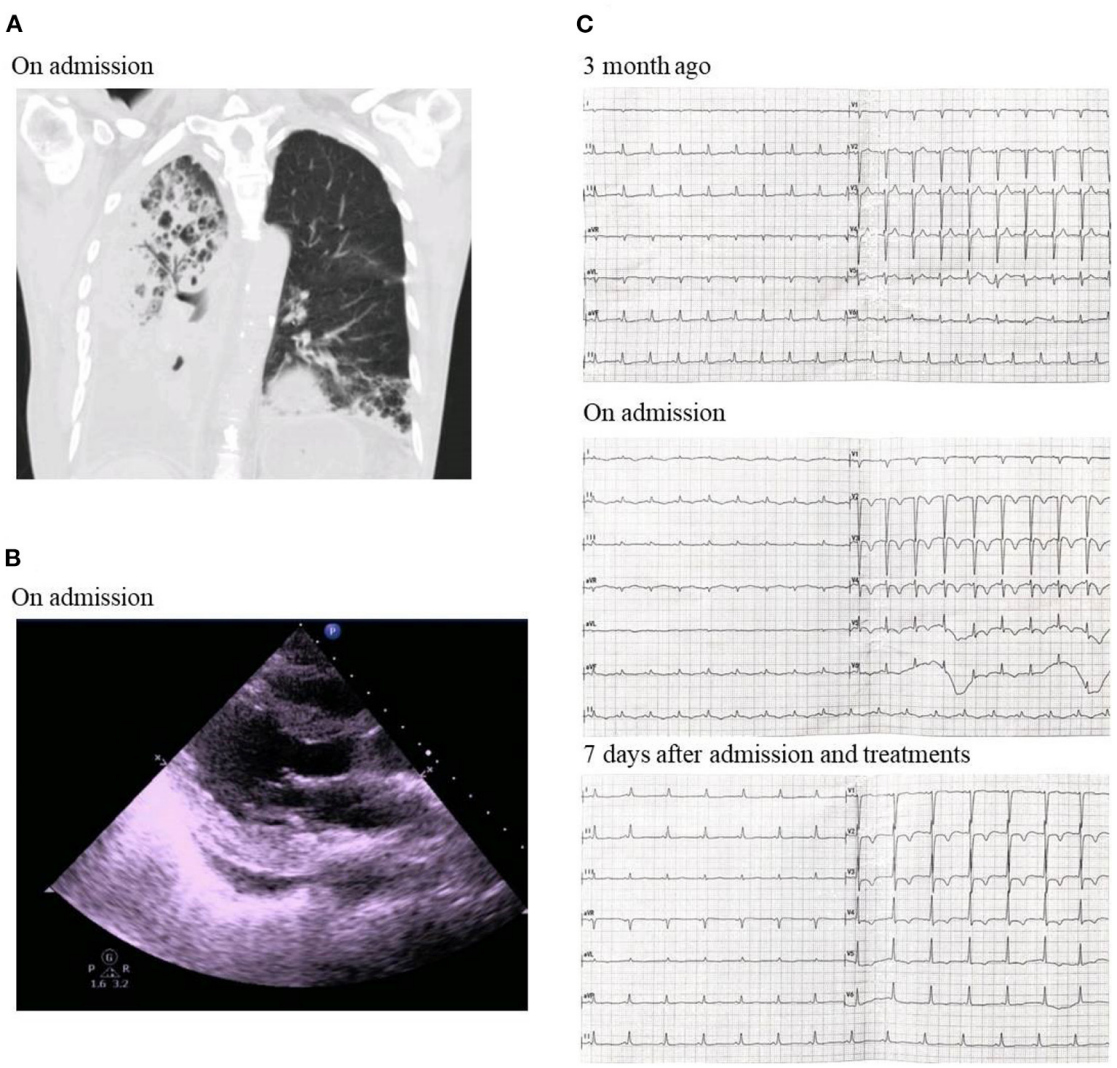
**(A)** Chest CT on admission. **(B)** Echocardiography showing ventricle size within normal range, increased atrium size and markedly decrease cardiac ejection fraction on admission. **(C)** Electrocardiogram showing sinus tachycardia, low voltage of limb leads, T wave inversion in anterior waves and V1–V3 QS type on admission.

Physical and laboratory examination was done for the patients upon this admission. The highest temperature was 39.4°C. His blood pressure was 121/69 mmHg. Chest computed tomography (CT) examination indicated right bronchial obstruction, obstructive pneumonia and right pleural effusion (Figure 1A). Echocardiography revealed ventricle size within normal range (left ventricle end diastolic dimension 46 mm), increased atrium size (left atrium dimension LA 36 mm) and markedly decrease cardiac ejection fraction (left ventricular ejection fraction 41%), tracing 2 mm pericardial effusion (Figure 1B). The electrocardiogram showed sinus tachycardia, low voltage of limb leads, T wave inversion in anterior waves and V1–V3 QS type (Figure 1C). Markers of myocardial injury were elevated: Natriuretic peptide BNP 18 942 ng/L; Troponin T 0.066 ng/L; Troponin I 200.83 ng/L; Creatine kinase (CK) and Creatine kinase isoenzyme (CKMB) normal. Moreover, inflammatory indicators were significantly elevated. Erythrocyte sedimentation rate (ESR) was markedly increased with the level of 101 mm/h, and C-reactive protein (CRP) 268.2 mg/L. Interleukin-6 was 44.93 pg/mL.

Judging by the decreased cardiac function and elevated myocardial injury markers at this admission, the patient was diagnosed of acute immune-associated myocarditis and right lung squamous cell carcinoma complicated with empyema. Treatments included methylprednisolone to suppress inflammation (40 mg, once per day, iv), meropenem to control infection (1.0 g, q8h, iv.drip) and symptomatic and supportive treatments. Seven days after admission, the patient's symptoms were relieved. Myocardial injury and inflammation markers were significantly decreased: Natriuretic peptide BNP was down to 2,298 ng/L; Troponin T, Troponin I, CK and CKMB normal; ESR 41 mm/h; CRP 29.8 mg/L; and Interleukin-6 normal. The electrocardiogram showed normal sinus rate and V2–V5 T wave inversion (Figure 1C). Echocardiography revealed ventricle size within normal range (left ventricle end diastolic dimension 48 mm), increased atrium size (left atrium dimension LA 30 mm) and markedly recovered cardiac ejection fraction (left ventricular ejection fraction 66%). The patient was discharged with prescription of continuing oral methylprednisolone (20 mg, once per day, po) and anti-bacterial therapy of faroenem to control infection (150 mg, q8h, po). No further heart failure exacerbations have occurred to date.

## Discussion

ICIs have substantially improved clinical outcomes in multiple cancer types (8, 9). Mechanistically, tumor cells realize immune escape by activating immune checkpoint molecular related signal pathway, and immune checkpoint inhibitors can awaken T lymphocytes to enhance the body's clearance of tumor cells. However, blocking immune checkpoints to restore antitumor immune response may also break immune tolerance to self-antigens and induce specific immune-related adverse events (10). With the widespread use of these drugs, immune related toxicity is increasingly recognized, including fatal myocarditis. Physicians should be aware of these infrequent, but potentially fatal toxicities associated with ICIs as their therapeutic use becomes widespread (11).

The ICI related myocarditis is generally considered highly arrhythmogenic and fatal; however, the exact pathogenesis is still poorly recognized and understood (12, 13). Myocarditis caused by ICIs represents <1% of immune-related adverse events. It is a potentially fatal condition associating with a mortality rate of 42% (14, 15). Judging by its high mortality rate, the incidence of ICI related myocarditis might be higher than expected. There are many manifestations of cardiotoxicity, such as myocarditis, heart failure, pericardial effusion, arrhythmia, acute coronary syndrome and valve disease. Treatment with ICIs can lead to severe and disabling inflammatory cardiovascular adverse-events soon after commencement of therapy (16). It is noteworthy that PD-L1 as monotherapy for lung cancer has been rarely reported to cause acute myocarditis. Previously it has also been reported of a patient diagnosed of non-small-cell lung cancer and developed durvalumab-associated myocarditis (17). As a result, it is worthy of further research and exploration whether there are differences in myocardial injury caused by different PD-L1 monotherapy and in patients with various baseline health state.

The keys to the diagnosis of ICIs related myocarditis in the present case are the previous history of PD-L1 utilization, elevated biomarkers suggesting cardiac damage, EKG presentation, negative coronary stenosis and decreased left ventricular ejection fraction.It is worthy of attention that in this case, PD-L1 monotherapy of durvalumab was chosen because of the right lung squamous cell carcinoma stage IV complicated with Prevotella nigrescens infection leading to empyema. In addition, due to the empyema, small dose of corticosteroids treatment was utilized in the present patient. Myocardial injury occurred in a short period and quickly returned to normal after treatment.

## Conclusion

In this study, we report a rare case of a 67-year-old male with lung squamous cell carcinoma complicated with empyema who experienced myocarditis after only PD-L1 inhibitor durvalumab monotherapy. As clinical evidence for myocarditis related to PD-L1 treatment has been limited, the present case report is of clinical value for the treatment and prognosis of PD-L1 related myocarditis.

## Data Availability Statement

The original contributions presented in the study are included in the article/supplementary material, further inquiries can be directed to the corresponding author/s.

## Ethics Statement

The studies involving human participants were reviewed and approved by The First Affiliated Hospital of Xi'an Jiaotong University. The patients/participants provided their written informed consent to participate in this study.

## Author Contributions

BZ and TC collected the clinical and laboratory data. JS and BZ summarized the data and drafted the manuscript. BZ, ML, and TC revised the final manuscript. All authors contributed to the article and approved the submitted version.

## Funding

This study was funded by Key Research and Development Program of Shaanxi (Program No. 2020KW-049).

## Conflict of Interest

The authors declare that the research was conducted in the absence of any commercial or financial relationships that could be construed as a potential conflict of interest.

## Publisher's Note

All claims expressed in this article are solely those of the authors and do not necessarily represent those of their affiliated organizations, or those of the publisher, the editors and the reviewers. Any product that may be evaluated in this article, or claim that may be made by its manufacturer, is not guaranteed or endorsed by the publisher.

## References

[1] Curti BD Faries MB. Recent advances in the treatment of melanoma. N Engl J Med. (2021) 384:2229–40. 10.1056/NEJMra2034861 34107182

[2] Xie X Wang X Liang Y Yang J Wu Y Li L. Evaluating cancer-related biomarkers based on pathological images: a systematic review. Front Oncol. (2021) 11:763527. 10.3389/fonc.2021.763527 34900711 PMC8660076

[3] Xiong A Wang J Zhou C. Immunotherapy in the first-line treatment of NSCLC: current status and future directions in China. Front Oncol. (2021) 11:757993. 10.3389/fonc.2021.757993 34900707 PMC8654727

[4] Wong SK Nebhan CA Johnson DB. Impact of patient age on clinical efficacy and toxicity of checkpoint inhibitor therapy. Front Immunol. (2021) 12:786046. 10.3389/fimmu.2021.786046 34868071 PMC8635107

[5] Di Wang KS Wang T Zhang D Sun F Cui Y Zhao H. Adverse effects and toxicity of immune checkpoint inhibitors for patients with urothelial carcinoma. Front Pharmacol. (2021) 12:710943. 10.3389/fphar.2021.710943 34867321 PMC8632774

[6] Lechner O Hu Y Jafarian-Tehrani M Dietrich H Schwarz S Herold M. Disturbed immunoendocrine communication via the hypothalamo-pituitary-adrenal axis in murine lupus. Brain Behav Immun. (1996) 10:337–50. 10.1006/brbi.1996.0030 9045749

[7] Zamami Y Niimura T Okada N Koyama T Fukushima K Izawa-Ishizawa Y. Factors associated with immune checkpoint inhibitor-related myocarditis. JAMA Oncol. (2019) 5:1635–7. 10.1001/jamaoncol.2019.3113 31436802 PMC6707099

[8] Wang DY Salem JE Cohen JV Chandra S Menzer C Ye F. Fatal toxic effects associated with immune checkpoint inhibitors: a systematic review and meta-analysis. JAMA Oncol. (2018) 4:1721–8. 10.1001/jamaoncol.2018.3923 30242316 PMC6440712

[9] Palaskas N Lopez-Mattei J Durand JB Iliescu C Deswal A. Immune checkpoint inhibitor myocarditis: pathophysiological characteristics, diagnosis, and treatment. J Am Heart Assoc. (2020) 9:e013757. 10.1161/JAHA.119.013757 31960755 PMC7033840

[10] Anquetil C Salem JE Lebrun-Vignes B Johnson DB Mammen AL Stenzel W. Immune checkpoint inhibitor-associated myositis: expanding the spectrum of cardiac complications of the immunotherapy revolution. Circulation. (2018) 138:743–5. 10.1161/CIRCULATIONAHA.118.035898 30359135

[11] Ball S Ghosh RK Wongsaengsak S Bandyopadhyay D Ghosh GC Aronow WS. Cardiovascular toxicities of immune checkpoint inhibitors: JACC review topic of the week. J Am Coll Cardiol. (2019) 74:1714–27. 10.1016/j.jacc.2019.07.079 31558256

[12] Li C Bhatti SA Ying J. Immune checkpoint inhibitors-associated cardiotoxicity. Cancers. (2022) 14:1145. 10.3390/cancers14051145 35267453 PMC8909315

[13] Power JR Alexandre J Choudhary A Ozbay B Hayek S Asnani A. Electrocardiographic manifestations of immune checkpoint inhibitor myocarditis. Circulation. (2021) 144:1521–3. 10.1161/CIRCULATIONAHA.121.055816 34723640 PMC8567307

[14] Hu JR Florido R Lipson EJ Naidoo J Ardehali R Tocchetti CG. Cardiovascular toxicities associated with immune checkpoint inhibitors. Cardiovasc Res. (2019) 115:854–68. 10.1093/cvr/cvz026 30715219 PMC6452314

[15] Chan KK Bass AR. Autoimmune complications of immunotherapy: pathophysiology and management. BMJ. (2020) 369:m736. 10.1136/bmj.m736 32253223

[16] Salem JE Manouchehri A Moey M Lebrun-Vignes B Bastarache L Pariente A. Cardiovascular toxicities associated with immune checkpoint inhibitors: an observational, retrospective, pharmacovigilance study. Lancet Oncol. (2018) 19:1579–89. 10.1016/S1470-2045(18)30608-9 30442497 PMC6287923

[17] Maetani T Hamaguchi T Nishimura T Marumo S Fukui M. Durvalumab-associated late-onset myocarditis successfully treated with corticosteroid therapy. Intern Med. (2022) 61:527–31. 10.2169/internalmedicine.7644-21 34433717 PMC8907760

